# Engineered Energy-Harvesting Hybrid Nanoscintillators for Enhanced Cancer Radiotherapy

**DOI:** 10.1021/acsami.6c02336

**Published:** 2026-03-06

**Authors:** Valeria Secchi, Irene Villa, Samuela Sala, Alessandro Colombo, Stefania Garbujo, Miriam Colombo, Angelo Monguzzi

**Affiliations:** † Department of Materials Science, 9305 Milano-Bicocca University, Via R. Cozzi 55, Milano 20125, Italy; ‡ NANOMIB, Center for Biomedical Nanomedicine, 9305 Milano-Bicocca University, P.Zza Ateneo Nuovo 1, Milano 20126, Italy; § Department of Biotechnology and Biosciences, University of Milano-Bicocca, Piazza della Scienza 2, Milano, Milan 20126, Italy

**Keywords:** radiotherapy, scintillators, energy harvesting, radiosensitization singlet oxygen, nanomaterials

## Abstract

During treatment, ionizing radiation interacts with biological tissues, generating high-energy charges that diffuse through the medium, damaging cellular DNA, or inducing the formation of cytotoxic reactive oxygen species (ROS) via water radiolysis. To enhance and localize ROS production by improving the interaction with ionizing radiation and optimizing the harvesting and utilization of deposited energy, we designed and developed a multicomponent nanomaterial as a prototype for the creation of coadjutant agents aimed at improving the efficacy and safety of radiotherapy. The system consists of a dense biocompatible magnesium silicate nanotube core, which enhances interaction with ionizing radiation, decorated with a dual layer of conjugated photosensitizers for singlet oxygen and ROS generation. Thanks to this optimized architecture that boosts the harvesting of the energy deposited by the ionizing radiation, exposure to X-rays resulted in a dramatic increase of almost 2 orders of magnitude in singlet oxygen generation yield compared to previously studied systems. This was accompanied by an excellent glioblastoma cell-killing efficiency at low concentrations, thus, strongly supporting the proposed nanomaterial architecture as a model for the development of next-generation radiotherapy coadjutants.

## Graphical abstract

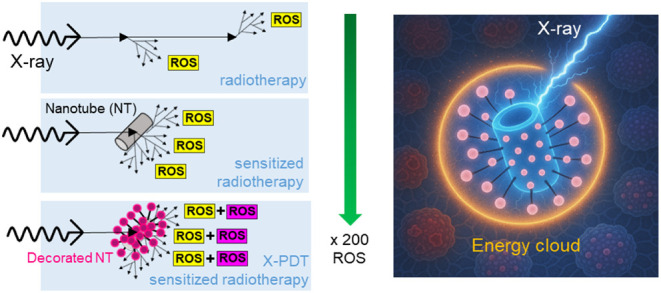

## Introduction

Radiotherapy (RT) is the most widely used clinical therapy pre- and postsurgery of deep-seated tumors. To kill the cancer cells, it exploits two different routes: direct DNA damage induced by the ionizing radiation and the indirect effect, due to the formation of cytotoxic reactive oxygen species (ROS), such as free radicals (hydroxyl radicals, hydrogen radicals, hydrogen peroxide, and hydroperoxyl radicals) and singlet oxygen (SO), upon radiolysis in the biological environment.^1^ Despite its effectiveness and frequent use, its application is still limited by the maximum dose of radiation that can be safely delivered to patients without significant side effects.^2^ Actually, the dose clinically provided (ca. 60 Gy total dose divided in daily fractions of 2–3 Gy) by using X-rays is generally not sufficient for a full effective treatment.^3–5^ The most advanced versions of this technique envisage the use of patient-specific dose-delivery plans to limit side effects or the use of short but highly intense radiation pulses;^6–8^ however, these approaches do not solve the problem of the lack of selectivity of the high-energy photons for the tumor tissues. Indeed, the high energies required to hit the tumors at a depth of a few centimeters in the body can also affect healthy tissues during their travel toward the tumor (Figure 1a).^9^

**Figure 1:**
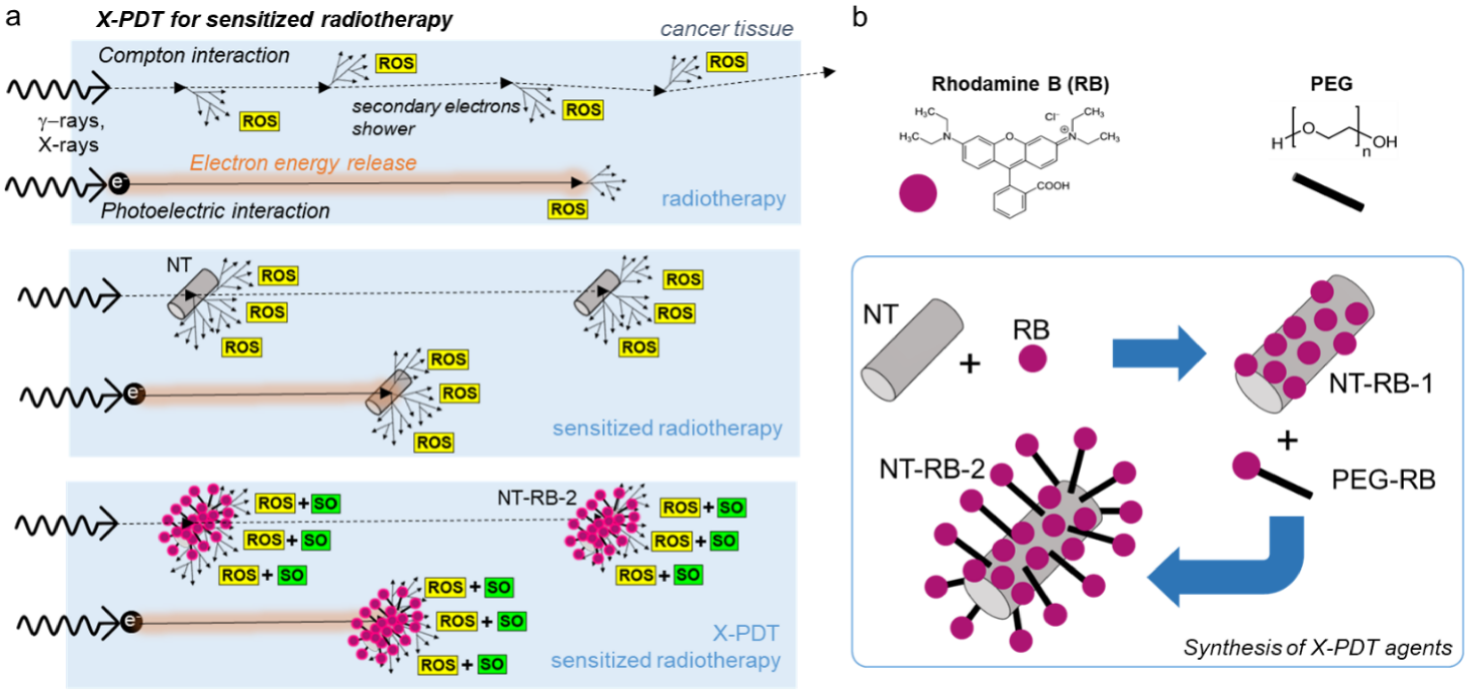
(a) Sketch of the working principles of radiotherapy that (top) produce cytotoxic reactive oxygen species (ROS) in the biological medium through water radiolysis. Black arrows indicate the electron diffusion within the tissue, and dashed arrows mark the photons’ path, even outside the sick tissue. (middle) Radiotherapy can be sensitized by using dense nanotubes (NTs) to enhance a localized release of the ionizing radiation (γ-rays and X-rays) and hot electron energy. (bottom) Radiotherapy can be further enhanced by exploiting X-ray-activated photodynamic therapy (X-PDT)-sensitized radiotherapy (bottom), where cytotoxic singlet oxygen (SO) production is enhanced through scintillating photosensitizer dyes (RB, Rhodamine B) that harvest the deposited energy. (b) Synthesis strategy and molecular structure of the SO sensitizer RB dye and of the PEG spacer used to realize the single-layer (NT-RB-1) and double-layer (NT-RB-2) decorated nanotubes used as X-PDT agents.

An important step forward in this technique, currently evaluated in ongoing trials,^10–12^ is the use of dense nanoparticles to target the cancer cells. The preferential interaction of the X-rays with the dense and high atomic number (*Z*) elements contained in the nanoparticles results in a most effective and localized release of the radiation energy with respect to the bare biological aqueous environment by means of a more probable Compton and photoelectric interactions with the incident high-energy photons (from 100 keV to 20 MeV) and thanks to a larger stopping power for the produced secondary electrons.^13–15^ The dose released in the tumor is therefore enhanced by this localized radiosensitization effect, which generates a larger density of ionized charges in the nanoparticle surroundings, and therefore in the targeted diseased cells sensitizing the ROS production through water radiolysis (Figure 1b).^16,17^ In this context, the therapeutic potential of medium- to high-*Z* catalytic nanosystems and semiconducting nanoparticles has been revealed, as they simultaneously enhance X-ray energy deposition and radiocatalysis capability.^5,18,19^ The generation of an elevated amount of free radicals in an aqueous environment, or the decomposition of H_2_O_2_ overexpressed within the tumor microenvironment into hydroxyl radicals by a nonoxygen-dependent radiocatalytic reaction, could increase ROS generation and induce DNA damage, thereby increasing the beneficial effects of radiotherapy. In recent years, ROS-enhanced nanomedicines mediated by nanoparticles have been studied for their ability to modulate intracellular redox balance with therapeutic anticancer effects. Notably, single-component high-*Z* nanosystems have attracted attention as vehicles to inhibit deep-seated tumors, even under hypoxic conditions, and potentially treat metastatic tumors.^20–24^

A further improvement of the radiosensitization is achieved by coupling the dense nanoparticles to photodynamic therapy (PDT)-active conjugated chromophores.^25,26^ The PDT is based on the employment of photosensitizer (PS) molecules, which are selectively activated by light in the presence of oxygen to produce ROS by charge (free radicals) and energy transfer processes (singlet oxygen), thus inducing the tumor death.^27,28^ The PDT has several advantages over conventional approaches, for example, it has no long-term side effects when correctly used, and it can also destroy the vasculature associated with the tumor, greatly contributing to the death of diseased cells.^29^ Unfortunately, deep tumors cannot be treated with PDT due to the poor penetration of the visible-NIR photons required to activate the best PS chromophores.^30,31^ The first attempt to overcome the poor penetration of light was proposed by Hashiguchi et al. in 2002, combining the use of the photosensitizer (PS) acridine orange with radiation therapy.^32^ Significant improvements in oncological medical trials have been achieved by developing hybrid systems in which photosensitizers are encapsulated in biocompatible carriers or chemically bonded to nanoparticles (Figure 1a).^33^ In these configurations, the PS can better harvest the energy released by the ionizing radiation and more efficiently capture secondary chargers, thus further sensitizing localized ROS production. The use of this X-ray-activated PDT (X-PDT) allows us therefore to exploit the PDT also in deep tissues. The synergistic use of radiosensitization and X-PDT effects, coupled to the short lifespan of many ROS species,^34^ gives a promising improvement of the therapy efficacy at a lower imparted dose, with a consequent reduction of the damages to healthy tissues. Since from when this concept was first proposed by Chen in 2006,^35^ it has undergone more than a decade of development both *in vitro* and *in vivo*.^25,36,37^ However, despite the good results obtained, it is still necessary to point out the best architecture of these multicomponent X-PDT systems that maximize the ROS sensitization. This is particularly true in clinical MV irradiation; here, the arrangement of PS around the dense multicomponent nanosystems becomes decisively important to trigger PS-photosensitized cytotoxicity in synergy with the augmented dose deposition.

Considering that the energy transfer from the dense nanoparticle to the PS molecules has a marginal role in the global energy partitioning process,^38^ we propose here an engineered X-PDT system based on a biocompatible magnesium silicate nanotube, as a dense radiosensitizer, decorated with a double layer of Rhodamine B (RB) molecules, one of the most effective PS available (Figure 1b).^39,40^ In such a way, we realized a highly efficient energy-harvesting network of PS around the nanotube, which allows to capture the lower energy electrons escaping from the dense core upon primary and secondary interactions under X-rays, as well as efficiently catch the ionized charges diffusing in the aqueous medium during thermalization. This particular design allows to enhance the radiosensitization effect of SO production by more than 1 order of magnitude with respect to the previously obtained systems and to the parent single-layer functionalized nanotubes, as well as an improved global cytotoxicity.^38^

## Results and Discussion

The multicomponent X-PDT system has been realized by coupling biocompatible hydrated magnesium silicate (Mg_3_(Si_2_O_5_)­(OH)_4_) nanotubes (Supporting Figure S1) with the RB molecules. The nanotubes have been prepared in an aqueous environment through a hydrothermal synthesis (Methods). They show a diameter of 30 nm and an average length of 100 nm (Figure 2a and Figure S2), i.e., optimal dimensions that favor the cellular uptake^41–43^ and allow the escape of ionized charges during irradiation.^16^ Their external surface is brucitic, and it tends to concentrate Mg^2+^ ions, allowing the Coulombic interactions with anionic species.^44–46^ Therefore, we exploit the RB carboxylic functional group to anchor the PS molecules directly on the nanotube surface (NT-RB-1). The RB amount has been chosen to be low enough to avoid intermolecular interaction between adjacent dyes and to leave some space for further functionalization with an optically inert spacer to realize a second layer of PS around the nanotube. In this case, we use as a spacer the polyethylene glycol (PEG). The employment of PEG is advantageous because it is an FDA-approved molecule with an anionic carboxylic function to anchor it to the cationic nanotube surface. Therefore, by using the RB-functionalized version of the spacer (PEG-RB, see Methods), we can effectively realize a second PS layer at a distance of ca. 4.6 nm from the nanotube core (NT-RB-2, Figure 1b).^47^ The functionalization of the nanotube surface with PS molecules is confirmed by vibrational and optical spectroscopy experiments. Figure 2b reports the infrared spectra of the bare and functionalized nanotube series. All spectra show the main nanotube vibrational peaks located around 3700 cm^–1^ (MgOH stretching) and in the region around 1000 cm^–1^ (Si–O–Mg, Si–O–Si, and Si–O stretching).^38^ The spectra of both NT-RB-1 and NT-RB-2 show the presence of peaks in the region around 2000 cm^–1^ related to the stretching of RB’s aromatic ring. In the NT-RB-2 spectrum, in addition to the RB features, we can observe the PEG absorption fingerprints of C=C stretching vibrations at 1634 cm^–1^, C=O stretching at 1734 cm^–1^, and a broad peak at 3416 cm^–1^ related to the hydroxyl group. Figure 2c reports the absorption spectra of the dispersions of the functionalized nanotubes in aqueous solution. The RB characteristic absorption peak around 565 nm is evident in both spectra, which allows to calculate the average number of RB molecules ⟨*n*⟩ attached on each nanotube. By considering the molar extinction coefficient ε of the RB and PEG-RB (Figure S3), we can calculate the total concentration of dye in the solution. Then, considering a nanotube with density 2.53 g/cm^3^, length 100 nm, inner diameter 7 nm, and outer diameter 30 nm, it is possible to assess their amount in the dispersion. We therefore obtain a ⟨*n*⟩ of ∼20 for the NT-RB-1 sample and ∼270 for the NT-RB-2 sample, respectively (Supporting Information, Section 2, Table S1). Considering the surface area of the nanotubes, these numbers correspond to a dye surface density of 0.04 dye/nm^2^ for NT-RB-1 and 0.6 dye/nm^2^ for NT-RB-2. The multistep functionalization allows therefore to increase by 1 order of magnitude in the density of dyes around the nanotube core, thus realizing a significantly denser energy-harvesting network.

**Figure 2:**
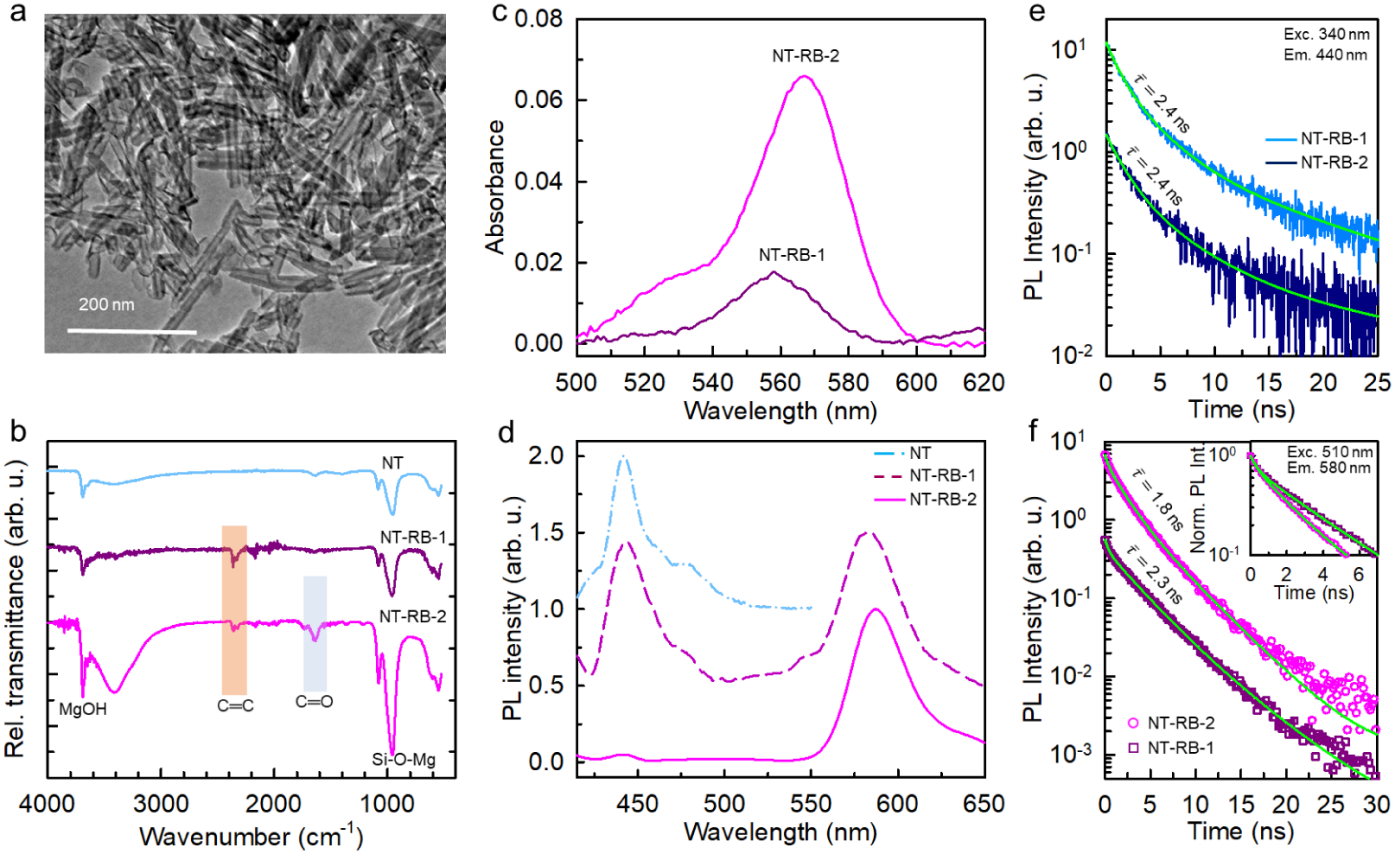
(a) Transmission electron microscopy (TEM) image of the bare nanotubes (NTs). (b) Attenuated reflectance FT-IR spectra of NTs, single-layer (NT-RB-1), and double-layer (NT-RB-2) NTs decorated with RB. (c, d) Absorption spectra (c) and photoluminescence (PL) spectra (d) under excitation at 350 nm of the NT series investigated in aqueous suspension (1 mg/mL). (e) PL intensity decay in time recorded at 430 nm under pulsed excitation at 340 nm for NT-RB-1 and NT-RB-2 samples. (f) PL intensity decay in time recorded at 580 nm under pulsed excitation at 510 nm for RB, NT-RB-1, and NT-RB-2 samples. Solid lines are the fit of data with multiexponential decay functions with characteristic decay time τ.

Prior to assessing the SO sensitization ability of the prepared multicomponent systems, we investigated their luminescence properties under UV excitation. As shown in Figure 2d, bare nanotubes show the typical weak emission in the blue spectral range peaked at 430 nm, while the usual RB emission at 580 nm can be clearly distinguished in both the functionalized nanotube samples. The photoluminescence shape of grafted RB is identical to that of one of the single molecules in diluted solution, thus suggesting the absence of emitting aggregates or undesired excimer species. No changes in the optical and emission properties appear after keeping functionalized nanotubes at 4 °C for up to 1 year after preparation, thus demonstrating the excellent stability of the nanomaterials (Figure S4. Time-resolved photoluminescence experiments shed more light on the electronic properties of the functionalized nanotubes. Figure 2e shows the nanotube emission intensity decay at 440 nm for NT-RB-1 and NT-RB-2 samples, respectively. No changes are observed in the RB-decorated samples, in agreement with the negligible energy transfer from the weakly emitting nanotube to the PEG-RB conjugated head, in agreement with the PEG spacer length.^48^ Nevertheless, the energy transfer from the dense core plays a negligible role in the X-PDT mechanism, so this will not reduce the system’s performance under X-rays.^38^ Figure 2f shows the emission intensity decay at 580 nm under direct excitation of the RB molecules at 510 nm. In the NT-RB-1 sample, we observe a slight initial acceleration of the emission (20% of the total, Supporting Table S2), which suggests a partial quenching of the excited state population for the dyes in close proximity to the nanotube surface, while most of the photons (80% of the total) are emitted with a lifetime of about 2.3 ns. This value is larger than the 1.7 ns of the isolated molecule (Figure S5) suggesting that the close proximity to the high refractive index nanotube surface slightly affects the oscillator strength of the dye radiative transition. Nevertheless, the mostly single exponential decay of the emission intensity and the matching of the photoluminescence spectrum with that of isolated RB molecules suggest that the anchored dyes preserve their excited state properties needed for the SO sensitization.^49^ On the other hand, the emission intensity of the NT-RB-2 sample shows a slightly multiexponential decay behavior, which hints at the presence of a distribution of locally diverse environments for the emitters. This can be due for example to weak intermolecular interactions between the conjugated heads of too close PEG-RB molecules. However, the photoluminescence average decay time is 1.8 ns, a value that matches that of the RB and of the PEG-RB moieties (Figure S5). This demonstrates that, globally, the PS dyes on the second functionalization layer are sufficiently far from the nanotube surface to be effectively considered as independent emitters and therefore fully operative as SO sensitizers. The proposed architecture for an optimized X-PDT system is therefore twice as effective, enabling (i) the loading of a much larger number of PS molecules on the same radiosensitizer nanotube with (ii) fully preserved ROS sensitization properties.

Figure 3a reports the radioluminescence spectrum of the nanotube series under exposure to soft X-rays. Confirming the photoluminescence experiments, the functionalized system shows the clear emission from the RB dyes at 580 nm, in addition to the nanotubes’ intrinsic blue emission at 430 nm. No significant emission can be observed in pure RB samples, thus confirming the dense nanotubes’ radiosensitization effect that better stops the X-rays from activating the conjugated chromophores (Figure S6). Notably, the RB vs nanotube relative emission intensity is almost doubled in the NT-RB-2 samples with respect to the NT-RB-1 system. This happens despite the lower number of nanotubes in the NT-RB-2 powder sample (Supporting Information, Section 2), thus pointing out the better energy-harvesting properties of the double-functionalized system that compensate the weaker interaction with the ionizing radiation. In order to evaluate the ROS sensitization efficiency of the different materials, we measured the relative efficiency of the SO sensitization in PBS dispersions, by using the SO sensor green (SOSG, Figure S7) as an optical probe, whose fluorescence is proportional to the concentration of SO.^50^ The SO generation upon irradiation has been monitored in the sample series for an exposure time of up to 500 s, which corresponds to a delivered dose of approximately 250 Gy (Methods). Figure 3b shows the obtained results, which have been normalized by the concentration of nanotubes in the solution. Bare nanotubes show a very little increment in the SO generation at short times with respect to the reference sample. Using the NT-RB-1 system, we observe a quick SO production at short times, as well as an increment of the final relative SO concentration about three times. Remarkably, using the NT-RB-2 system, we observe a total increment of the SO concentration of about 60 times higher with respect to the NT-RB-1 system and 200 times higher with respect to bare nanotubes, i.e., 2 orders of magnitude, which clearly points out the ameliorated performances of the proposed material as an efficient energy harvester and SO sensitizer, in agreement with the radioluminescence experiments discussed above.

**Figure 3:**
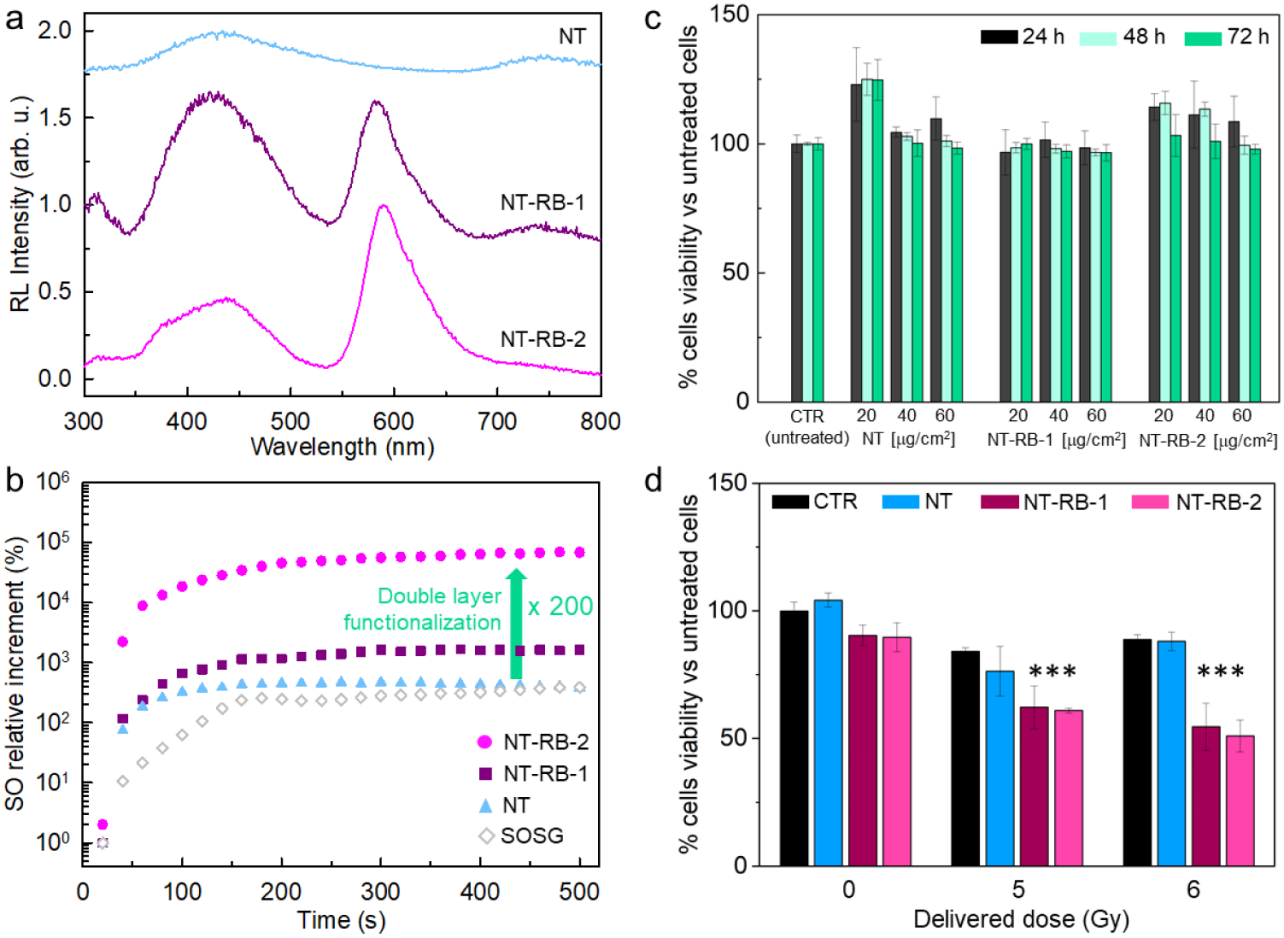
(a) Radioluminescence (RL) spectra of bare nanotubes (NTs), NT-RB-1, and NT-RB-2 sample powders under soft X-ray exposure. (b) Relative increment of the SO concentration as a function of the X-ray irradiation time for the NT sample series (1 mg/mL, PBS dispersion). (c) Evaluation of cell viability by the MTS test on U-87 cells incubated with 20, 40, and 60 μg cm^–2^ of NT, NT-RB-1, and NT-RB-2 after 24, 48, and 72 h. Results shown as % cell viability normalized vs untreated sample, mean with standard deviation (*n* = 6). Statistical analysis: two-way ANOVA, *p* < 0.0001 (***). (d) Evaluation of the X-ray irradiation effect immediately after irradiation by the MTS test on U-87 cells incubated with 20 μg cm^– 2^ of NT, NT-RB-1, and NT-RB-2 samples.

The biocompatibility and X-PDT activity of the bare and functionalized nanotubes were evaluated by means of the MTS assay on a human glioblastoma U-87 cell line (Methods). This line is the most commonly validated model for a deadly brain tumor and, importantly, it is known for its radioresistance.^51–54^ Therefore, this cell line is ideal to test the proposed radiotherapy coadjutants.

Figure 3c shows the results of the biocompatibility test performed as a function of the nominal concentration used to treat the cells. The viability of the cell population incubated with nanotubes has been checked at 24, 48, and 72 h after seeding checkpoints. The histogram reveals that using concentrations of 40 and 60 μg/cm^2^, the cellular proliferation is slowed down with respect to the unstained control culture after the same incubation time using NT-RB-1 or NT-RB-2, with a viability reduction of ∼10% in both cases. Conversely, using a concentration of 20 μg/cm^2^, we observe a negligible decrease of less than 5% in cell viability after 72 h, thus indicating the absence of nonspecific cytotoxicity. With this treatment, the effect of the irradiation on U-87 cells has been monitored by exposing the cells to soft X-rays with a delivered dose of up to 6 Gy (Figure 3d). No improvement in the cell death rate is observed for the population stained with bare NTs. Conversely, the incubation with NT-RB-1 and NT-RB-2 induces a dramatic drop in the cell population, which in both cases is halved at 6 Gy of delivered dose.^55^ Importantly, it is worth recalling that at the fixed concentration of 20 μg/cm^2^ used for the staining, the total amount of nanotubes in the NT-RB-2–treated sample is −43% less than in the NT-RB-1–treated sample. These findings further confirm the enhanced X-PDT ability of the double-layer-decorated NT-RB-2 system, where the proposed optimized architecture allows achieving a better cytotoxic effect despite a significantly lower amount of radiosensitizers. This result suggests that the more efficient energy-harvesting network of PS molecules in the NT-RB-2 system allows better exploitation of the deposited energy to generate more and more localized SO, pointing out a crucial role of the structural design of multicomponent dense nanomaterials to obtain more effective radiotherapy coadjutants.

In order to clarify the cellular death mechanisms involved, we performed additional experiments. First, we evaluate the effect of nanotubes on the direct cellular DNA damage by monitoring the DNA double-strand breaks induced by the ionizing radiation (Methods, Figures S8–S13). As reported in Figure S13, the fraction of DNA-damaged cells increases with the delivered dose and, in agreement with the higher SO production that enhances also DNA damage, we observe the highest fraction of damaged cells in the presence of the best SO sensitizer NT-RB-2.^56^ Following radiotherapy, the resulting DNA double-strand breaks typically lead to reduced clonogenicity within days or weeks rather than immediate cell death. Since we observed a significant change in cell viability in the presence of NT-RB-1 and NT-RB-2 with X-PDT immediately after irradiation (Figure 3d), we decided to investigate the cause of X-PDT-induced cell death at relatively short times using an apoptotic/necrotic assay through Annexin V-FITC staining after 24 h from irradiation, i.e., after a single round of cell division (Methods, Figure S14).^57,58^ The results are summarized in Figure 4.

**Figure 4:**
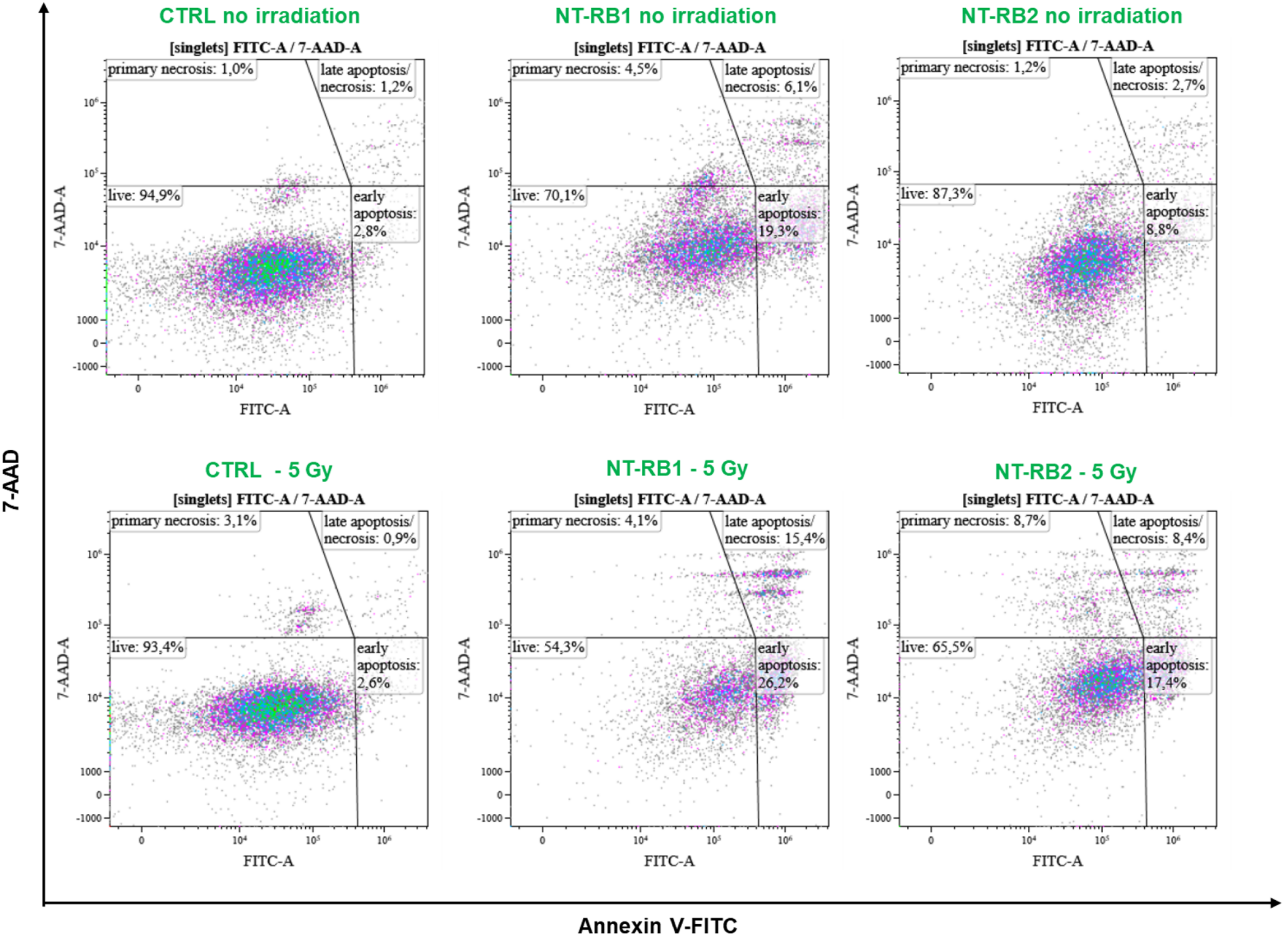
Flow cytometry assessment of cell death in U-87 cells (Annexin V/7-AAD). Representative flow cytometry dot plots of U-87 cells stained with Annexin V-FITC and 7-AAD. Untreated control cells (CTRL) and cells treated with NT-RB-1 or NT-RB-2 were analyzed under nonirradiated conditions or 24 h after irradiation (5 Gy). Quadrant gating identifies live (Annexin V^–^/7-AAD^–^), early apoptotic (Annexin V^+^/7-AAD^–^), late apoptotic/necrotic (Annexin V^+^/7-AAD^+^), and primary necrotic (Annexin V^–^/7-AAD^+^) populations. Percentages of each population are reported in the corresponding panels.

The data confirm a higher biocompatibility of the NT-RB-2 system as the fraction of viable cells in the absence of irradiation (87.3%) remains close to that of the untreated control (94.5%). Conversely, the presence of NT-RB-1 directly causes a drop in the viable cell population down to 70.1% induced mainly by early apoptosis. Upon irradiation (5 Gy), both nanostructured systems induced marked changes in the distribution of cell populations compared to their nonirradiated counterparts. NT-RB-1–treated cells exhibited a reduction in the live population down to 54.3%, concomitant with a significant increase in early apoptotic cells (26.2%) and late apoptotic/necrotic cells (15.4%). Similarly, NT-RB-2 treatment led to a decrease in live cells down to 65.5%, with early apoptosis accounting for 17.4% and late apoptosis and necrosis accounting for 8.4% of the total population. Overall, at 24 h postirradiation, NT-RB-1– and NT-RB-2–treated cells exhibited comparable levels of cell death mainly associated with apoptotic pathways, particularly early apoptosis, while late apoptosis/necrosis contributed to a smaller extent. Again, this happens despite the total amount of nanotubes in the NT-RB-2–treated sample being halved with respect to the NT-RB-1–treated sample, thus demonstrating the enhanced X-PDT ability of the NT-RB-2 system.

## Conclusion

To summarize, we successfully developed a novel multicomponent scintillating nanomaterial, specifically designed as an optimized X-PDT agent and intended to serve as an effective coadjutant for radiotherapy. The system structure consists of a dense nanotube core, functionalized with two layers of scintillating conjugated dyes that act as photosensitizers for generating singlet oxygen, which is a well-known ROS that is essential for tumor cell eradication. Our multistep functionalization strategy enables the stable anchoring of hundreds of photosensitizer molecules onto the nanotube surface while preserving their photophysical properties, which are critical to their function. The resulting system demonstrates a significantly enhanced capacity to produce singlet oxygen within the cellular environment and exhibits a strong therapeutic effect under X-ray exposure—exceeding previously investigated systems by more than 1 order of magnitude. Considering the low density of the employed dyes, which does not affect the nanotube’s ability to interact with X-rays affecting water radiolysis, the observed improvement can be ascribed to the enhanced ability of the optimized material to harvest the energy deposited by the ionizing radiation and produce cytotoxic effects through singlet oxygen sensitization. These results highlight a general design strategy for maximizing energy harvesting and optimizing the use of deposited radiation energy in biological tissues—especially important in scenarios where the concentration of dense radiosensitizers must be minimized due to cellular uptake limitations, systemic delivery constraints, potential adverse effects from suboptimal biodistribution, and, possibly, to balance the hypoxic conditions typical of tumor tissues that limit the radiotherapy effect. Considering the high biocompatibility of the employed nanotubes, the next step toward a possible clinical validation will involve the fabrication of this nanomaterial using components already approved by regulatory agencies, including clinically approved photosensitizers, to facilitate a faster transition to in vivo testing and potential therapeutic applications.

## Methods

### Synthesis of Nanotubes (NTs)

Nanotubes were synthesized according to a previously used synthetic method.^30^ A hydrothermal reactor with a 100 cm^3^ polypropylene vessel was used to carry out the hydrothermal reaction of 1522 mg of Na_2_SiO_3_ and 764 mg of MgCl_2_ in an aqueous solution of NaOH (220 mL, 0.4 M) at 250 °C with a run duration of 16 h. The precipitate removed for the solution was repeatedly washed with deionized water before being dried for 3 h at 110 °C.

### Functionalization of Nanotubes with Rhodamine B

60 mg of NT’s powder were dispersed in 15 mL of PBS, and 2 mL of Rhodamine B solution (3 × 10^–5^ M in PBS) were added at the dispersion under stirring for 10 min. Samples were centrifuged for 5 min at 6500 rpm. The precipitate removed for the solution was repeatedly washed with deionized water before being dried for 3 h at 50 °C.

### Double Functionalization of NTs with Rhodamine B and Rhodamine B-PEG2k-COOH

60 mg of NT-RB-1 were dispersed in 15 mL of PBS, and 2 mL of Rhodamine B-PEG2k-COOH solution (2 mg/7 mL PBS) were added at the dispersion under stirring for 10 min. Samples were centrifuged for 5 min at 6500 rpm. The precipitate removed for the solution was repeatedly washed with deionized water before being dried for 3 h at 50 °C

### Diffraction Experiment (XRD)

Powder XRD patterns were acquired in Bragg–Brentano geometry with Cu Kα radiation (Analytical X’Pert Pro powder diffractometer).

### Transmission Electron Microscopy

Transmission electron microscopy (TEM) observations were performed with a JEOL JEM1220. TEM samples were prepared by dispersing a few milligrams of the compounds in 2 mL of distilled water and dropping 3 μL of the solution on carbon-coated copper grids.

### ATR-FTIR (Attenuated Total Reflection Fourier Transform Infrared Spectroscopy)

ATR-FTIR spectra of dried samples were obtained on a Thermo Scientific Nicolet iS20 FTIR Spectrometer.

### Optical Studies

Absorption spectra were recorded by using a Cary Lambda 900 spectrophotometer at normal incidence with Suprasil quartz cuvettes with a 1 cm optical path length. Steady-state PL and PLE spectra have been recorded using a xenon lamp as an excitation source, together with a double monochromator (Jobin-Yvon Gemini 180 with a 1200 grooves/mm grating), and recorded through a nitrogen-cooled CCD detector coupled to a monochromator (Jobin-Yvon Micro HR). Under continuous wave laser excitation, signals were recorded using a nitrogen-cooled CCD coupled with a double monochromator, Triax-190 (HORIBA Jobin-Yvon), with a spectral resolution of 0.5 nm.

### Time-Resolved Photo/Radioluminescence

Time-resolved PL spectra were recorded using a pulsed LED at 340 nm (3.65 eV, EP-LED 340, Edinburgh Instruments, with a pulse width of 700 ps) or a pulsed laser at 510 nm (3.06 eV, EPL-405, Edinburgh Instruments, with a pulse width of 150 ps). Data were obtained with an Edinburgh Instruments FLS-980 spectrophotometer with a 5 nm bandwidth and a time resolution of 0.1 ns.

### Soft X-ray Radioluminescence

RL excitation was obtained by X-ray irradiation through a Be window, using a Philips 2274 X-ray tube with a tungsten target operated at 20 kV (average energy 7 keV). RL measurements were carried out on powders at room temperature using a homemade apparatus featuring, as a detection system, a charge-coupled device (CCD) (Jobin-Yvon Spectrum One 3000) coupled with a spectrograph operating in the 200–1100 nm range (Jobin-Yvon Triax 180). The data were corrected for the spectral response of the detection system. RL spectra were recorded in powder filled sample holders with 1 cm diameter and 0.1 thickness.

### Singlet Oxygen Relative Concentration Measurement

The relative singlet oxygen production was performed under continuous X-ray exposure at 7 keV (dose rate 27.6 mGy/mA*s with uncertainty 2%) in a set of dispersions made of Singlet Oxygen Sensor Green (SOSG) as a fluorescent optical probe in PBS and nanotubes or the functionalized nanotubes. SOSG powder was dissolved in a 1:10 solution of DMSO and PBS, while the final dispersions were at a 1 mg/1 mL concentration). During the X-ray irradiation for a total of 500 s, the increment of singlet oxygen concentration was monitored by exciting the SOSG fluorescence by a continuous wave laser at 473 nm and by observing the progressive increase of the probe luminescence intensity at 530 nm.

### Cell Culture

Human primary glioblastoma cells U-87 MG were purchased from ATCC (HTB-14) and were cultured in high-glucose Dulbecco’s Modified Eagle’s Medium (DMEM) supplemented with 10% fetal bovine serum (FBS), 2 mM l-glutamine, penicillin (50 IU mL^–1^), and streptomycin (50 mg mL^–1^). The cell line was maintained at 37 °C in a humidified atmosphere containing 5% CO_2_ and passaged before confluence. Cells were regularly tested for mycoplasma, and all experiments were performed on mycoplasma-free cells.

### Cell Viability

Cells were seeded in a 96-multiwell plate at 3000 cells/well. The following day, cells were incubated with NTs, NT-RB-1, and NT-RB-2 at concentrations of 20, 40, and 60 μg/cm^2^ (respectively, equivalent to 64, 128, and 192 μg/mL, considering the surface area and volume of the wells), for 24 h, 48 h, or 72 h. At the end of the incubation period, the NT-containing medium was removed, cells were washed with sterile PBS, and fresh medium was added to the wells (100 μL/well). 20 μL of MTS solution were added to every well, in accordance to manufacturer’s instructions, and 490 nm absorbance was read after 3 h of incubation with a PerkinElmer multimodal plate reader. Results are shown as % cell viability normalized vs untreated sample, mean with standard deviation (*n* = 6).

### Viability Tests after Irradiation

U-87 MG cells were seeded in 35 mm tissue culture-treated Petri dishes (Corning, vacuum gas plasma-treated) at a density of 30,000 cells per dish in 1.5 mL of complete medium. After 24 h, the culture medium was replaced with 1 mL of fresh complete medium supplemented with 20 μg cm^–2^ of NT, NT-RB-1, and NT-RB-2, while untreated cells served as controls. Cells were further incubated for 24 h at 37 °C and subsequently exposed to X-rays at nominal doses of 0, 5, or 6 Gy. Immediately after irradiation, cells were rinsed with PBS and incubated for 3 h at 37 °C in 800 μL of fresh DMEM supplemented with 200 μL of MTS stock solution [3-(4,5-dimethylthiazol-2-yl)-5-(3-carboxymethoxyphenyl)-2-(4-sulfophenyl)-2*H*-tetrazolium]. Following incubation, 100 μL of the resulting violet formazan-containing solution was transferred in triplicate to a 96-well plate. Absorbance was recorded at 490 nm using an EnSight multimode plate reader, and cell viability was determined by normalizing the treated sample values to those of the untreated controls.

### Immunofluorescence Analysis for DNA Damage Characterization

U-87 cells were seeded in 48-well plates containing 12 mm glass coverslips precoated with Cultrex. Cells were plated at a density of 2.0 × 10^4^ cells per well in 0.5 mL of complete culture medium. After 24 h, cells were treated with NT-RB-1 and NT-RB-2 at a surface concentration of 10 μg cm^–2^. 24 h after treatment, cells were exposed to X-ray irradiation (5 or 6 Gy). Immediately after irradiation, the culture medium was replaced with fresh complete medium, and cells were reincubated for 2 h at 37 °C in a humidified atmosphere with 5% CO_2_. The medium was then gently removed, and cells were briefly rinsed with 200 μL per well of 1× PBS containing Ca^2+^/Mg^2+^ without incubation. Cells were fixed with 250 μL per well of 4% paraformaldehyde (PFA) for 15 min at room temperature in the dark, ensuring complete coverage of the coverslips. After fixation, PFA was removed, and cells were washed twice with 200 μL per well of cold PBS (Ca^2+^/Mg^2+^) for 5 min at room temperature. Cells were then permeabilized with 250 μL per well of 0.2% Triton X-100 in PBS for 5 min at room temperature, followed by blocking with 5% fetal bovine serum (FBS) in PBS (250 μL per well) for 1 h at room temperature in the dark. Cells were incubated overnight at 4 °C with an anti-phospho-histone H2A.X (Ser 139) primary antibody (mouse monoclonal, Santa Cruz Biotechnology, cat. no. sc-517348), diluted 1:400 in PBS. After washing with PBS (Ca^2+^/Mg^2+^) for 5 min at room temperature, cells were incubated for 1 h at room temperature in the dark with an Alexa Fluor 647–conjugated antimouse secondary antibody (Thermo Fisher Scientific, cat. no. A-21235), diluted 1:1000 in PBS. Cells were washed with PBS and counterstained with Hoechst (5 μg mL^–1^; Thermo Fisher Scientific, cat. no. 662249) for 5 min at room temperature, followed by a final PBS wash. Coverslips were then carefully removed, briefly rinsed in Milli-Q water, and mounted onto ethanol-cleaned glass slides using 5 μL of FluorSafe mounting reagent. Slides were allowed to dry for 10 min, incubated at 37 °C for 20 min, and stored at −20 °C until imaging. As a positive control for DNA double-strand breaks, cells were treated with etoposide (1–10 μM) for 1 h in complete culture medium prior to fixation. The percentage of cells showing DNA double-strand breaks over the total number of seeded cells was counted starting from the analyses in Figures S9–S11. A standard deviation was calculated over a triplicate of independent measurements.

### Flow Cytometry Analysis

U-87 cells were seeded in 100 mm diameter Petri dishes at a density of 6.0 × 10^4^ cells per dish in 1.5 mL of complete culture medium. After 24 h, cells were treated with NT-RB-1 and NT-RB-2 at a surface concentration of 10 μg cm^2^. Following an additional 24 h of incubation, cells were exposed to X-ray irradiation (5 Gy), after which the culture medium was replaced with fresh complete medium and cells were incubated at 37 °C in a humidified atmosphere with 5% CO_2_. 24 h postirradiation, cells were detached using trypsin, collected, and washed twice with cold PBS. For the positive control, cells were heat-treated at 65 °C for 10 min to induce late apoptosis (Figure S13). Cells were resuspended in 100 μL of Annexin V binding buffer containing Annexin V-FITC (Thermo Fisher Scientific, A13201) and incubated for 5 min at room temperature in the dark. Subsequently, 5 μL of 7-AAD (BioLegend, 420404) was added, followed by an additional 10 min of incubation in the dark. After staining, 50 μL of binding buffer was added and samples were immediately analyzed by flow cytometry using an Aurora Cytek instrument. Cell populations were classified as live (Annexin V^–^/7-AAD^–^), early apoptotic (Annexin V^+^/7-AAD^–^), late apoptotic/necrotic (Annexin V^+^/7-AAD^+^), and primarily necrotic (Annexin V^–^/7-AAD^+^), and the percentage of each population was quantified.

## Supplementary Material



## Notes

Additional data on structural characterization of nanotubes and optical and photoluminescence properties; analysis of time-resolved photoluminescence and scintillation; analysis of powder radioluminescence; data on radiosensitization experiments and DNA experiments (DOCX)
