# Supplementary material for: Engineered Energy-Harvesting Hybrid Nanoscintillators for Enhanced Cancer Radiotherapy
Source: ACS Appl Mater Interfaces. 2026 Mar 6;18(10):14822–31. doi: 10.1021/acsami.6c02336 (PMC13006953; doi:10.1021/acsami.6c02336)
Supplement: Supplementary file 1 [file am6c02336_si_001.docx]

**Supporting Information for**

Engineered energy-harvesting hybrid nanoscintillators

for enhanced cancer radiotherapy.

*Valeria Secchi*^1,2^*, Irene Villa^1,2^, Samuela Sala^1^, Alessandro Colombo^2,3^, Stefania Garbujo^2,3^, Miriam Colombo^2,3^, Angelo Monguzzi*^1,2^*

*^1^ Department of Materials Science, Milano-Bicocca University, via R. Cozzi 55, 20125 Milano, Italy*

*^2^NANOMIB, Center for Biomedical Nanomedicine, Milano-Bicocca University, P.zza Ateneo Nuovo 1, 20126, Milano, Italy.*

*^3^Department of Biotechnology and Biosciences, University of Milano-Bicocca, Milano, Piazza della Scienza 2, 20126, Milan Italy.*

*Corresponding author email address:* [*angelo.monguzzi@unimib.it*](mailto:angelo.monguzzi@unimib.it)

**Table of contents**

1. Structural characterization of nanotubes………………………… page 2

2. Optical and photoluminescence properties……………………… page 3

3. Time resolved photoluminescence and scintillation analysis……. page 5

4. Radioluminescence analysis on powders………………………… page 6

5. Radiosensitization experiments. ………………………………….. page 6

6. DNA damage data. page 8

7. Supplementary References. ……………………………………….. page 13

1. **Structural characterization of nanotubes.**


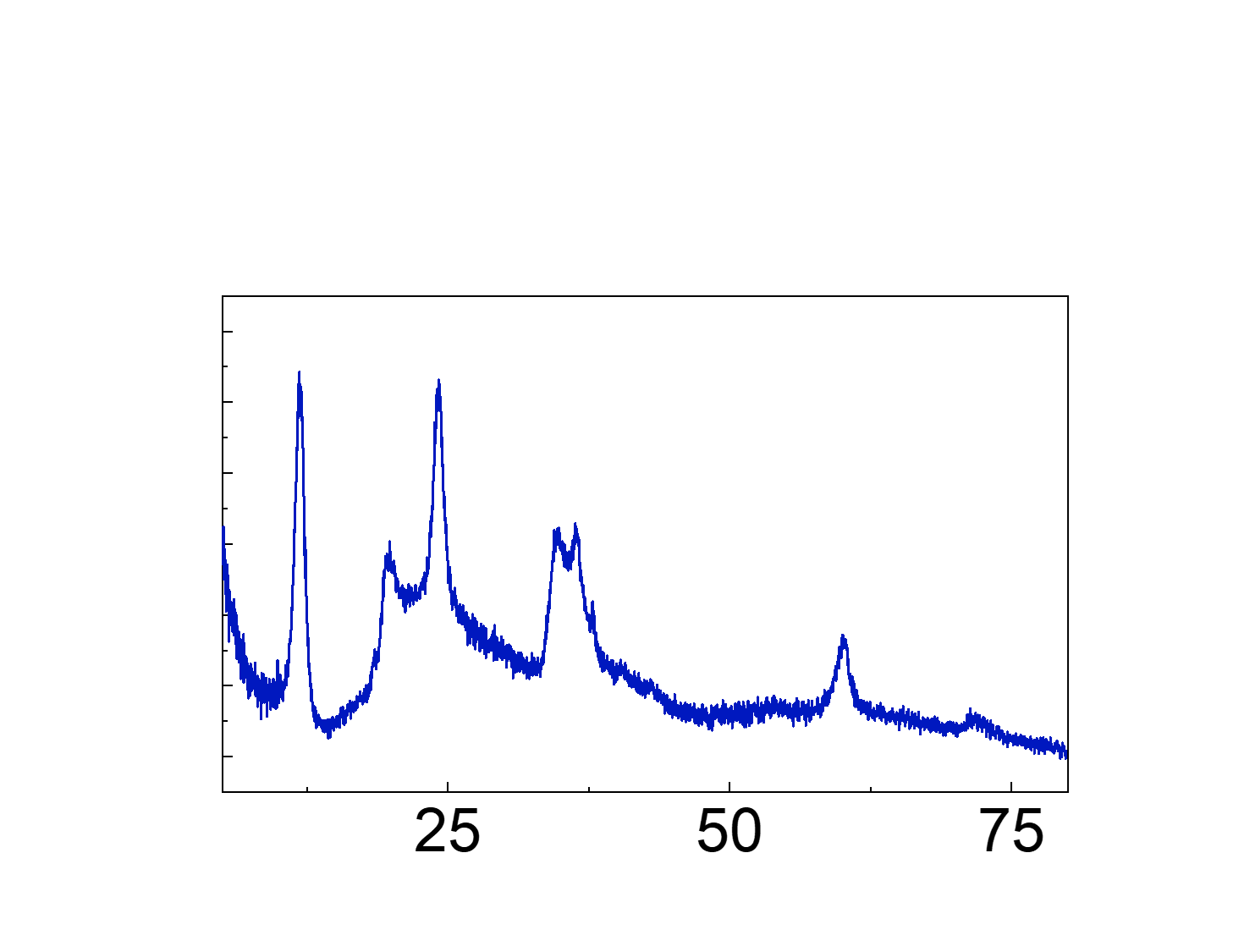


2θ°

Intensity (arb.u.)

NT

**Figure S1.** P-XRD patterns of the powder of bare hydrated magnesium silicate nanotubes in agreement with previous results .^1^


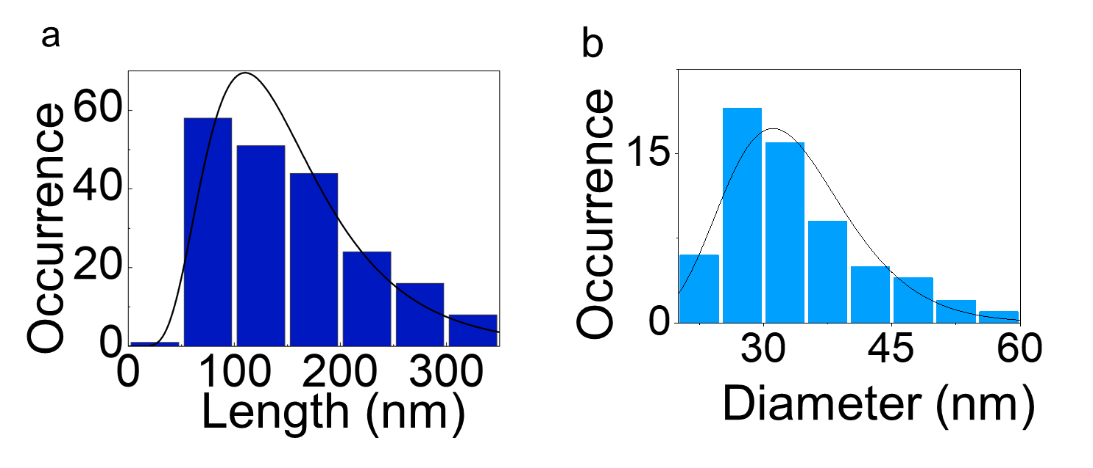


**Figure S2.** Nanotubes length (a) and outer diameter (b) distribution estimated from the analysis of the TEM images (Fig. 1). The solid black line is the fit of data with a log-normal distribution with mode of 100 nm for nanotubes length and 30 nm for the diameter.

1. **Optical and photoluminescence properties**

*2.1 Absorption properties of Rhodamine B (RB) and Rhodamine B-PEG2k-COOH (PEG-RB)*


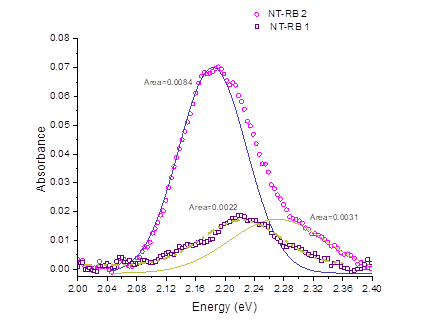


**Figure S3.** (left) Absorbance spectra and Extinction molar coefficient of Rhodamine B (RB) and Rhodamine B-PEG2k-COOH (PEG-RB) in aqueous solution.^2^ (right) Nanotubes absorbance spectra reported in energy (eV). Gaussian components (solid and dotted lines) obtained by the numerical fit (green dashed line) are shown together with experimental curves (open circles and squares) PL spectra were deconvolved into Gaussian components using the Levenberge-Marquardt algorithm and we considered the energy of the component in Rhodamine B E=2.22 -2.27 eV (546-558 nm) and the one in Rhodamine B-PEG2k-COOH E=2.18 eV (568 nm) while the FWHM were set in the range of 0.12 - 0.15eV. From the fitting of the absorption spectra of the multicomponent system we estimated the contribution of each component (RB and PEG RB). The molar extinction coefficient to be used was calculated from the absorption of Rhodamine B Molecule and Rhodamine B-PEG2k-COOH molecule obtaining ε (PEG-RB) = 26254 cm^-1^M^-1^ and ε (RB) = 95593 cm^-1^M^-1 .^

*2.2 Calculation of dye density on nanotubes and nanotube concentration.*

**Table S1.** Composition parameters for the NT series investigated. Considering the molecular weight of RB (479g/mol) and PEG-RB (2500 g/mol) and given the chrysotile density of 2.53 g/cm3, a tube geometry with average length 100 nm, inner diameter 7 nm, and outer diameter 30 nm, we can calculate the number of NTs /mg: 4.4x1012 NT-RB2 and 6.3x1012 NT-RB1.

| **Sample** | **Composition** | **Absorbance** | **ε (cm^-1^M^-1^)** | **#dye/NT** |
| --- | --- | --- | --- | --- |
| NT-RB 1 | 3.4 mg/3ml | 0.017 | 95593 | 20 |
|  |  |  |  |  |
| NT-RB 2 | 3.5 mg/3ml | 0.066 | 26254 | 240 |
|  |  | 0.016 | 95593 | 28 |

*2.3 Nanotubes stability vs time.*


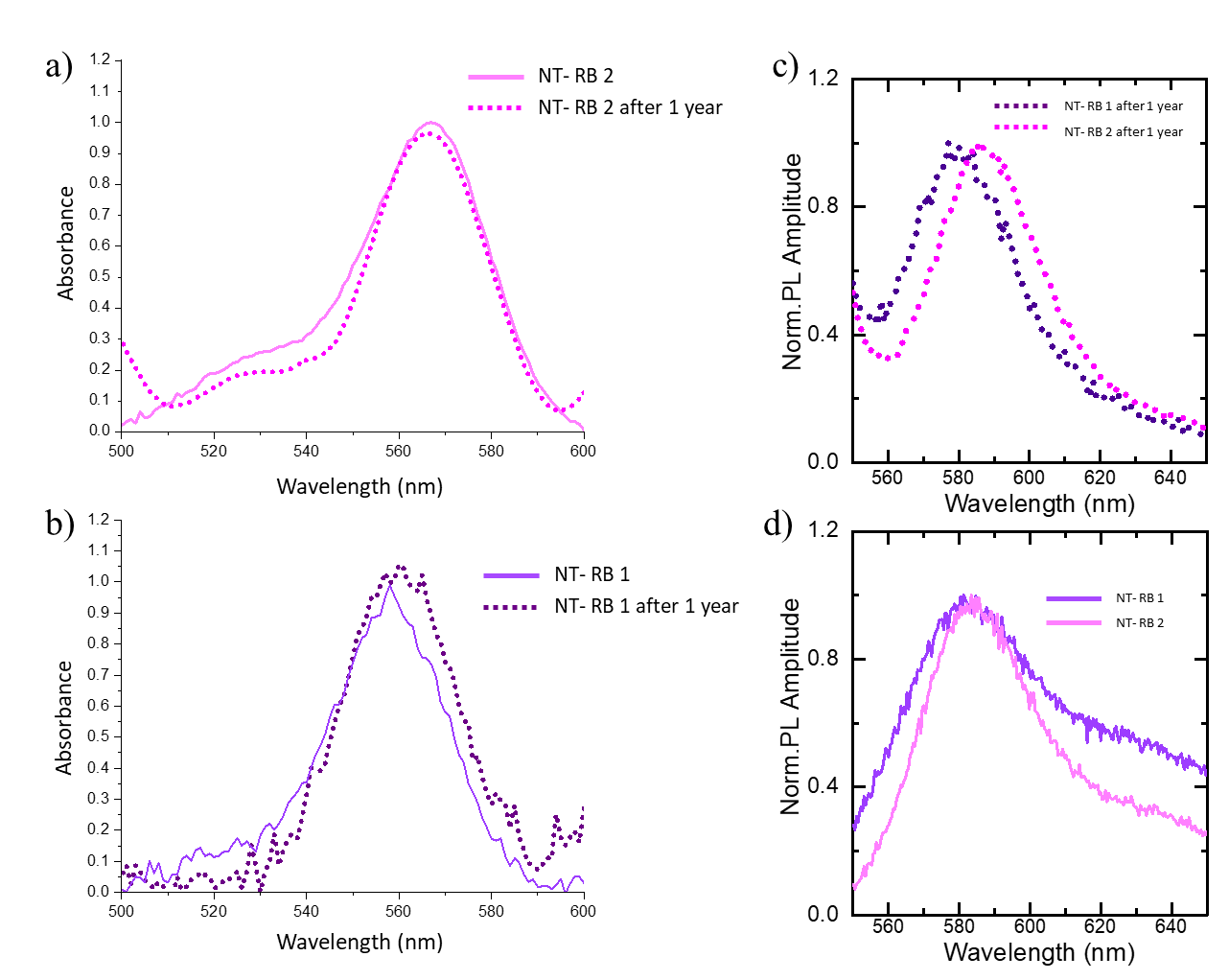


**Figure S4.** Absorbance (a, b) and photoluminescence under 540 nm excitation (c, d) of 1 mg/mL aqueous solution of nanotubes of fresh batches and after 1 year from the synthesis.

1. **Time resolved photoluminescence and scintillation analysis**

The time resolved photoluminescence and scintillation measurements described in the main text allow to monitor the decay in time of the luminescence intensity. The intensity decay is typically fitted using multi-exponential functions as

$I_{PL (scint)}\left( t \right)\propto\sum_{i} A_{i}e^{-(\frac{t}{\tau_{i}})}$ . Eq. S1

The average emission characteristic lifetime has been calculated as

$\bar{\tau}=\sum_{i} \frac{A_{i}\tau_{i}}{A_{i}}$. Eq. S2

**Table S2.** Fit parameters employed to reproduce the photoluminescence intensity decay at 50 nm under 510 pulsed excitation (Figs. 2, 2f)

| sample | **A_1_** | $\boldsymbol{\tau}_{\boldsymbol{1}}$ | **A_2_** | $\boldsymbol{\tau}_{\boldsymbol{2}}$ |
| --- | --- | --- | --- | --- |
| **NT-RB-1 @ 430 nm** | 0.84 | 1.93 ns | 0.16 | 11.55 ns |
| **NT-RB-2 @ 430 nm** | 0.86 | 1.90 ns | 0.14 | 11.79 ns |
| **NT-RB-1 @ 580 nm** | 0.20 | 0.43 ns | 0.80 | 3.57 ns |
| **NT-RB-2 @ 580 nm** | 0.35 | 0.65 nm | 0.65 | 2.89 ns |


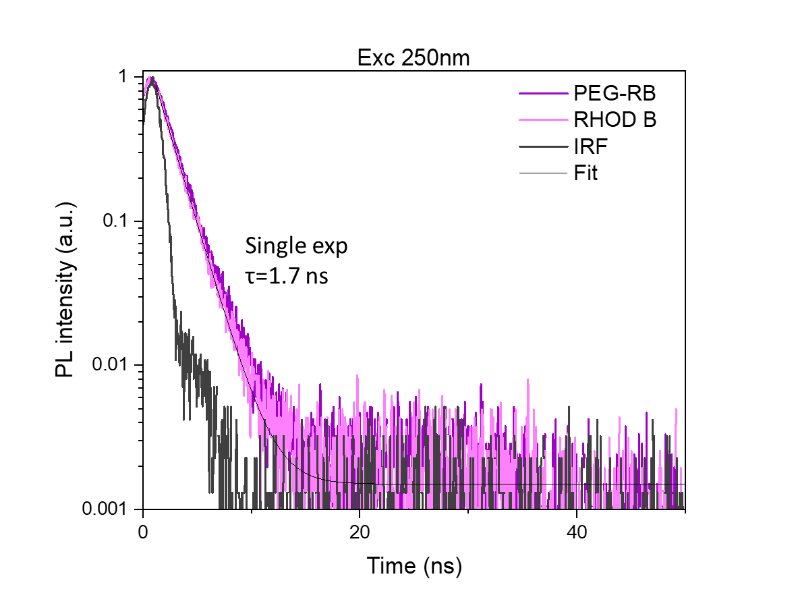


**Figure S5.** Time resolved photoluminescence decay of Rhodamine B (10^-5^M) and PEG-RB (4×10^-5^ M) in aqueous solution under 510 nm excitation. Instrumental response (IRF) is depicted together with the samples’ decays showing a single exponential behaviour with a lifetime of 1.7 ns. ^1^

1. **Radioluminescence analysis on powders.**

**Figure S6.** Radioluminescence spectra of Rhodamine B (RB) powders, NT- RB1 and NT- RB2 powders. The samples consist in 1 mg of powder deposited on aluminium plate. (Left) RL spectra of the samples normalized to the number of dyes. For a better visualisation the rhodamine B RL amplitude was multiplied by a factor of 2000. The RL red-shift of the dye emission in the bare rhodamine B powder with respect to the RB functionalized nanotubes depends on the aggregation of the molecules. (Right) RL spectra of NT- RB1 and NT- RB2 powders normalized to the NT emission revealing the larger sensitization of dye emission for NT- RB2. The spectra were acquired under 7 keV soft X-rays and corrected by the spectral sensitivity of the detector.

1. **Radiosensitization experiments.**

**Figure S7.** Normalized RL spectra of SOSG and of all the NT-based systems over the 25 measurements. Bare NT and functionalized NT have been added into a solution of SOSG (SOSG powder in 1:10 solution of DMSO and PBS). X-ray tube voltage of 20 kV has been applied in concomitant with *cw* laser excitation at 473 nm. The estimated dose for each measurement is of 10 Gy (nominal dose in air).

1. **Immunostaining for DNA damage monitoring.**

DNA damaging has been assessed through immunofluorescence experiments using Ab Phospho-Histone H2A.X + anti-mouse-647 (γ-H2AX) to label the double-strand breaks. Cell nuclei have been stained with Hoechst blue. Etoposide was used as a positive control to induce DNA damage.


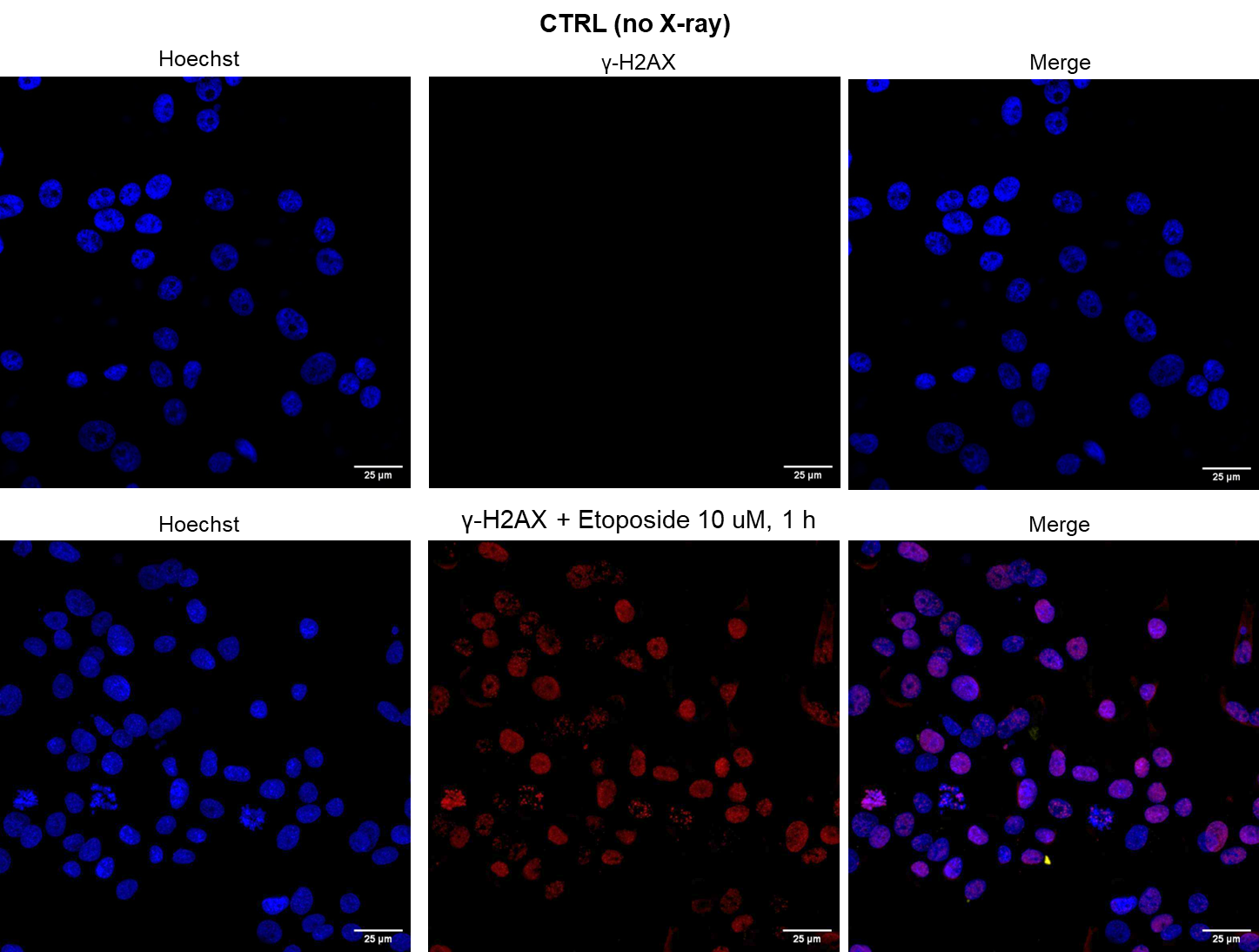


**Figure S8.** Control experiment for DNA damaging detection on untreated U-87 cells. Cell nuclei are shown in blue, while DNA double-strand breaks are identified in red by γ-H2AX immunostaining. Etoposide was used as a positive control to induce DNA damage and to verify the selective staining ability of γ-H2AX for DNA double-strand breaks. No irradiation has been used. Scale bar: 25 µm.


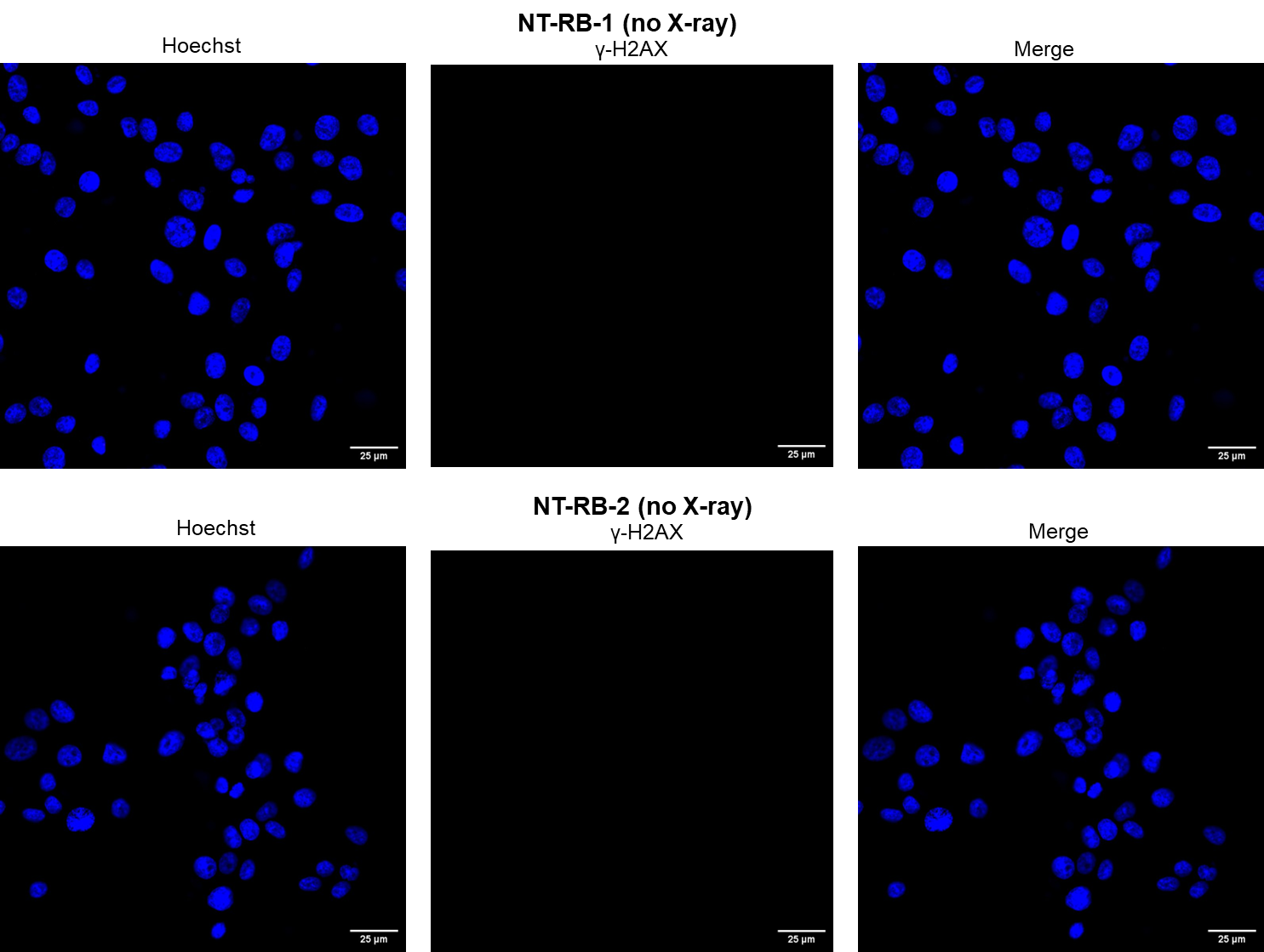


**Figure S9.** Control experiment for DNA damaging detection on treated U-87 cells without irradiation. Cell nuclei are shown in blue, while DNA double-strand breaks are identified in red by γ-H2AX immunostaining. No irradiation has been used. Scale bar: 25 µm.


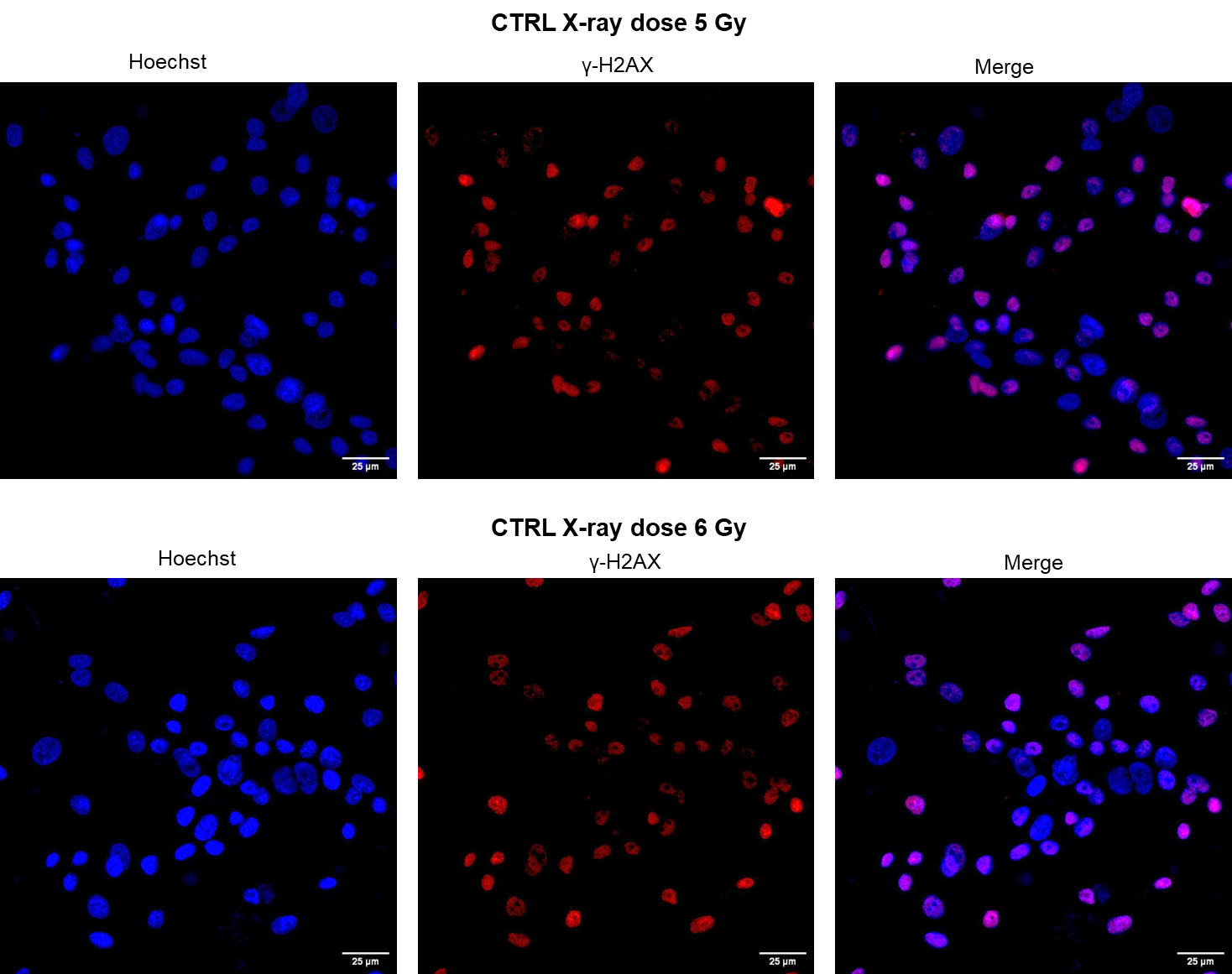


**Figure S10.** DNA damaging detection on U-87 cells at different delivered X-rays doses. Cell nuclei are shown in blue, while DNA double-strand breaks are identified in red by γ-H2AX immunostaining. Scale bar: 25 µm.

**
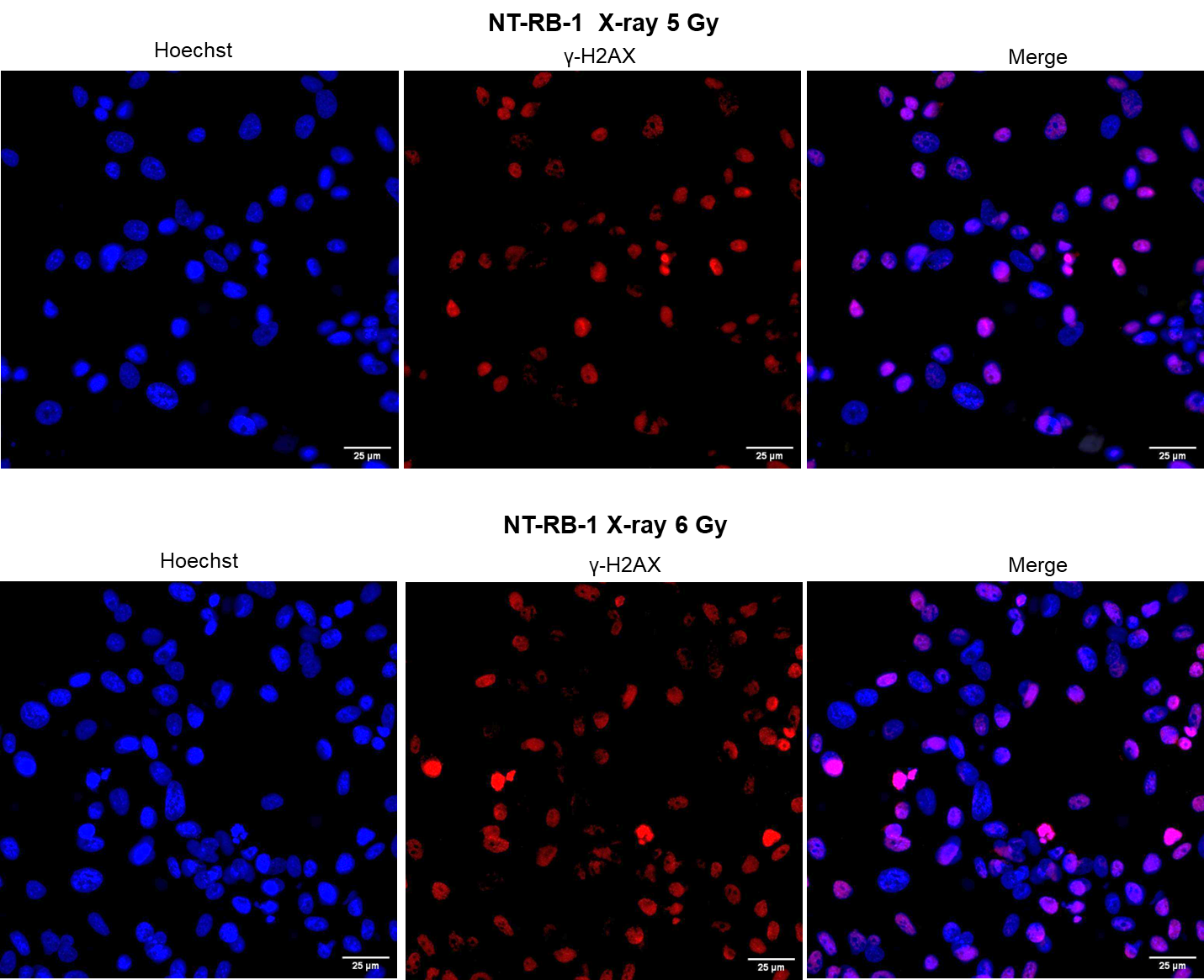
**

**Figure S11.** DNA damaging detection on U-87 cells incubated with NT-RB-1 (10 μg/cm^2^) at different delivered X-rays doses. Cell nuclei are shown in blue, while DNA double-strand breaks are identified in red by γ-H2AX immunostaining. Scale bar: 25 µm.


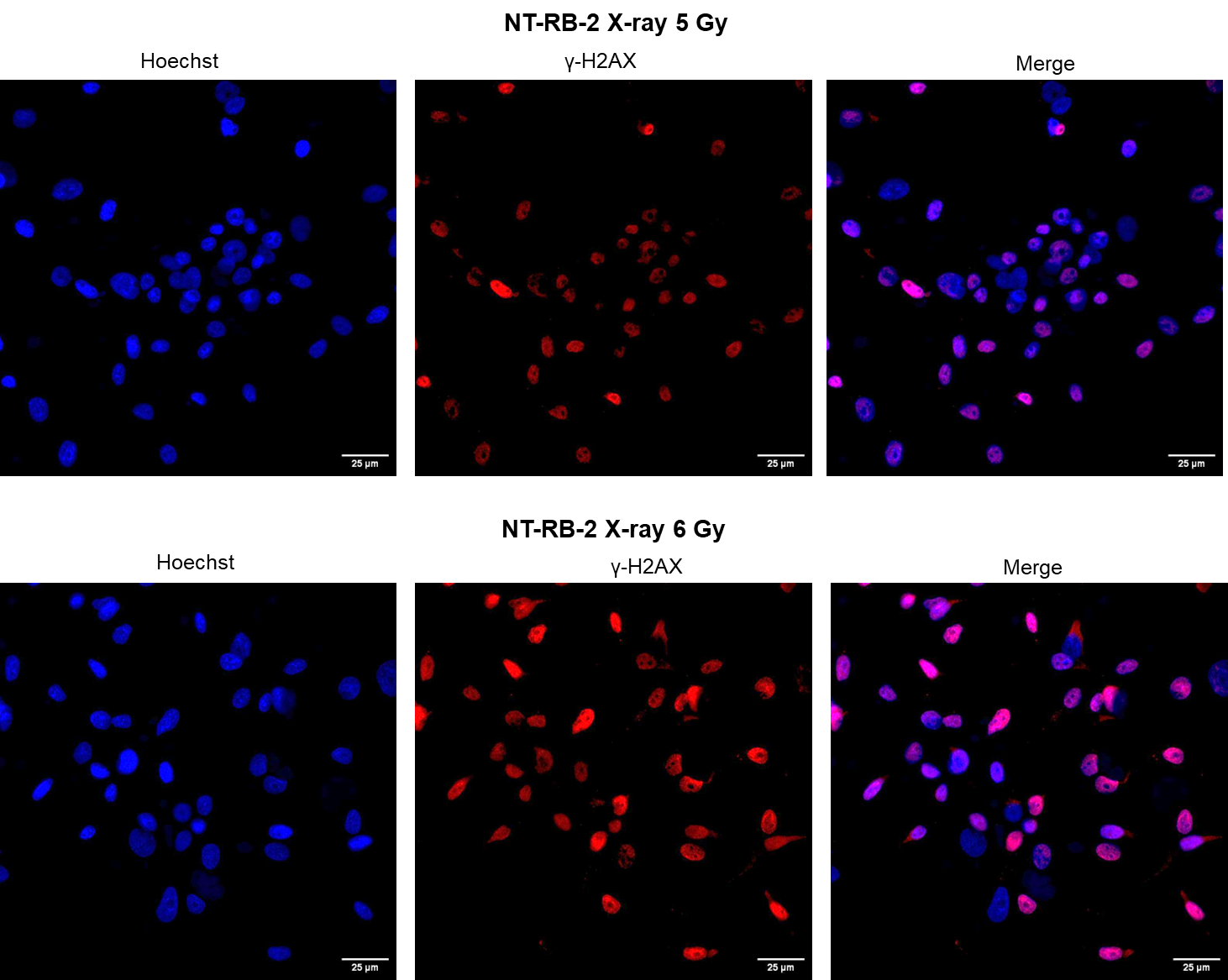


**Figure S12.** DNA damaging detection on U-87 cells incubated with NT-RB-2 (10 μg/cm^2^) at different delivered X-rays doses. Cell nuclei are shown in blue, while DNA double-strand breaks are identified in red by γ-H2AX immunostaining. Scale bar: 25 µm.

**Figure S13.** Quantification of DNA double-strand breaks in U-87 cells detected by γ-H2AX immunostaining. The percentage of γ-H2AX–positive cells is shown for untreated controls (CTRL) and cells treated with RB1 or RB2, following exposure to different X-ray doses (5 Gy and 6 Gy). Error bars represent the standard deviation from three independent replicates.

**
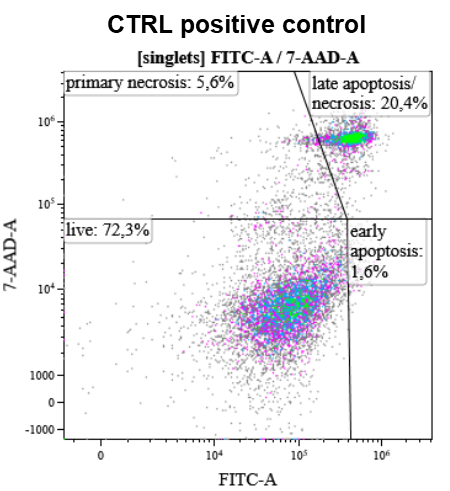
**

**Figure S14. Flow cytometry dot plot analysis of U-87 cells stained with Annexin V-FITC and 7-AAD.** This panel represents a positive control in which cells were heat-treated at 65°C for 10 minutes to induce late apoptosis.

1. **Supplementary References.**

(1) Secchi, V.; Cova, F.; Villa, I.; Babin, V.; Nikl, M.; Campione, M.; Monguzzi, A. Energy Partitioning in Multicomponent Nanoscintillators for Enhanced Localized Radiotherapy. *ACS Applied Materials & Interfaces* **2023**, *15* (20), 24693–24700. DOI: 10.1021/acsami.3c00853.

(2) Craparo, E. F.; Musumeci, T.; Bonaccorso, A.; Pellitteri, R.; Romeo, A.; Naletova, I.; Cucci, L. M.; Cavallaro, G.; Satriano, C. mPEG-PLGA Nanoparticles Labelled with Loaded or Conjugated Rhodamine-B for Potential Nose-to-Brain Delivery. *Pharmaceutics* **2021**, *13* (9), 1508.
